# Collective States, Multistability and Transitional Behavior in Schooling Fish

**DOI:** 10.1371/journal.pcbi.1002915

**Published:** 2013-02-28

**Authors:** Kolbjørn Tunstrøm, Yael Katz, Christos C. Ioannou, Cristián Huepe, Matthew J. Lutz, Iain D. Couzin

**Affiliations:** 1 Department of Ecology and Evolutionary Biology, Princeton University, Princeton, New Jersey, United States of America; 2 School of Biological Sciences, University of Bristol, Bristol, United Kingdom; 3 Cristian Huepe Labs Inc., Chicago, Illinois, United States of America; Tel Aviv University, Israel

## Abstract

The spontaneous emergence of pattern formation is ubiquitous in nature, often arising as a collective phenomenon from interactions among a large number of individual constituents or sub-systems. Understanding, and controlling, collective behavior is dependent on determining the low-level dynamical principles from which spatial and temporal patterns emerge; a key question is whether different group-level patterns result from all components of a system responding to the same external factor, individual components changing behavior but in a distributed self-organized way, or whether multiple collective states co-exist for the same individual behaviors. Using schooling fish (golden shiners, in groups of 30 to 300 fish) as a model system, we demonstrate that collective motion can be effectively mapped onto a set of order parameters describing the macroscopic group structure, revealing the existence of at least three dynamically-stable collective states; swarm, milling and polarized groups. Swarms are characterized by slow individual motion and a relatively dense, disordered structure. Increasing swim speed is associated with a transition to one of two locally-ordered states, milling or highly-mobile polarized groups. The stability of the discrete collective behaviors exhibited by a group depends on the number of group members. Transitions between states are influenced by both external (boundary-driven) and internal (changing motion of group members) factors. Whereas transitions between locally-disordered and locally-ordered group states are speed dependent, analysis of local and global properties of groups suggests that, congruent with theory, milling and polarized states co-exist in a bistable regime with transitions largely driven by perturbations. Our study allows us to relate theoretical and empirical understanding of animal group behavior and emphasizes dynamic changes in the structure of such groups.

## Author Summary

The patterns exhibited by moving animal groups like flocks of birds and schools of fish are typical of self-organizing systems in which global structural and dynamical properties arise from local interactions between individuals. Despite their apparent complexity, such systems can often be described, and understood, in terms of these emergent properties, rather than the detailed low-level description needed for depicting the individual dynamics. Here we show that schooling fish (in groups of 30 to 300 golden shiners) can be described in terms of the degree of alignment and degree of rotation among group members. We demonstrate that shiner schools exhibit three distinct behaviors: a swarm state with low speeds and little order; a strongly aligned state where the fish move with higher speeds; and a milling state, where each fish moves around the center of the group. Simulations have previously predicted this type of behavior, and we relate our findings to a well-known model of collective motion to help highlight similarities and differences between models and animal groups. Our results give insight into the regulation of group structure among animals, and also inform us generally about how global structures arise naturally from interactions among components of dynamical systems.

## Introduction

Many animal groups display coordinated motion in which individuals exhibit attraction towards others and also a tendency to align their direction of travel with near-neighbors [1]. The functional complexity of such aggregates are thought to result from relatively local, self-organizing interactions among individuals that endow many groups, such as flocking birds and schooling fish, with the capacity to move, respond to threats and make decisions collectively [2]. Thus the way in which collective dynamics emerge from inter-individual social interactions likely has profound consequences on the selection pressures experienced by group living organisms.

Theoretical considerations of the self-structuring properties of groups have suggested that certain features of interaction among individuals may give rise to a relatively small number of specific collective states [3]–[7]. For example, models representing local repulsion, directional alignment and longer-range attraction among individuals [3], [5] predict that groups in which individuals follow these behavioral rules exhibit only three collective states; a *swarm* state in which individuals aggregate but are locally and globally disordered, a *milling* state in which individuals have a high degree of alignment with local neighbors but overall the group exhibits a rotating milling formation (or torus, in three-dimensional space) and a *polarized* state in which individuals tend to be aligned with each other over a long range and consequently the group experiences net movement. While these patterns have all been observed in nature among different organisms [8] and can evolve in simulations of simple predator and prey behaviour [9], experimental support for the existence of these states, and for any one system transitioning between them, has not previously been determined.

In addition to displaying commonly observed group structures these models also emphasize an important unifying feature—that collectively moving animal groups can be considered as a dynamical system in which multiple group states, or dynamically-stable states, exist, and that collective properties, such as the spatio-temporal configurations exhibited, may be robust to exactly how behavioral tendencies such as repulsion, alignment and attraction are mediated. Furthermore, under this scenario, animal groups are also predicted to exhibit ‘multistability’ whereby more than one collective state coexist for identical individual behavior, with groups transitioning relatively quickly between the alternate structural configurations. In the model of Couzin et al. [3], for example, the milling and polarized state co-exist over a region of parameter space (see Paley et al. [10] for a formal analysis of this bistable regime). The question of which collective behavior is adopted therefore depends on the initial configuration of the system, in this case the positions and orientations of individuals: if groups start in a relatively disordered state they tend to form a milling formation; but tend to remain in the polarized configuration if they begin sufficiently aligned with one another. Perturbations, such as disruption induced by predator attacks [1], or more generally, sources of intrinsic [11], [12] or extrinsic noise [13], can cause the system to leave its existing dynamically-stable state and enter an unstable transitional regime. Depending on the degree and type of perturbation the group may find itself either drawn back towards the previous state, or if perturbed sufficiently far, to be drawn into the alternative dynamically-stable state.

Thus even though the group behavior results from a large number of relatively local interactions, the group-level dynamics can be described using relatively few and simple lower-dimensional ‘order parameters’ that portray the collective dynamics, such as global polarization and the degree of collective rotation. This approach is familiar to us in physical systems where a multitude of different inter-molecular interactions result in only four fundamental states of matter; solid, liquid, gas and plasma. When properties such as density and energy are altered, a physical system can undergo ‘phase transitions’ between these states. Similarly, at a certain level of description, we can view animal groups as having the potential to exhibit abrupt changes in spatial or temporal patterns, and thus phase transition-like behavior (see Buhl et al. [14] for an experimental example of density-driven transitions in locust swarms).

However, biological systems are not in equilibrium. There are no conserved thermodynamic quantities, such as momentum and energy, and individual motion typically results not from thermal fluctuations or external forcing, but from individual self-propulsion and decision-making. Nevertheless, the concept that local interactions reduce to common non-equilibrium collective states is a key insight provided by computational modeling of collective animal behavior [3], [4], [15] which relates more generally to phase transition theory in non-equilibrium systems [16]. Additionally, despite individual motion likely being governed by a complex stochastic decision-making process based on the positions, movement and size of neighbors (and even hidden features like individual's state), two recent studies of schooling fish—Katz et al. [17] and Herbert-Read et al. [18]—demonstrate that interactions can be effectively reduced to local tendencies to be repelled from, or attracted towards, neighbors. Similar evidence exists for aggregating ducks [19] and swarming locusts [20].

Despite these advances, to date, no experimental study has quantified the dynamical states of collective motion exhibited by any species of group-living animal, nor determined whether the general predictions of existing models of collective behavior hold—notably, that groups will exhibit, and transition among, relatively few (dynamically-stable) states. Here we investigate the emergence of macroscopic collective states under highly controlled laboratory conditions using schooling fish (golden shiner, *Notemigonus crysoleucas*). This is a convenient model system for investigating collective behavior since individuals are relatively small (average length approximately 5 cm in our study), and naturally form highly cohesive schools in very shallow and still water [21]. Digital tracking of fish in a range of group sizes (from 30 to 300 fish) allows us to obtain detailed data regarding the individual positions and velocities of schooling fish over long periods of time. We use these data to analyze how group size and perturbations (driven both by inevitable contact with the boundary of the tank, but also by changes in motion by individuals within the group in the absence of boundary influence) affect group behavior and function to transition the group between alternative dynamical states.

## Results/Discussion

Seven replicate experiments were conducted for group sizes 30, 70 and 150 fish, and three replicates were conducted for 300 fish (due to limitations in our capacity to house very large numbers of fish). Each replicate consisted of filming fish swimming in a large shallow tank (2.1 m×1.2 m, water depth 5 cm) for 56 minutes (at 30 frames per second). A similar approach has been taken previously [17], [18], [22], [23]. Individual fish were tracked following the methodology of Katz et al. [17] to obtain time series of the positions and velocities. These time series constitute the raw data from which we base our analyses.

To describe the collective structure of the fish shoals we use two order parameters, identical to previous categorization in simulation models [3], [11]. First, the polarization order parameter *O_p_*, which provides a measure of how aligned the individuals in a group are. It is defined as the absolute value of the mean individual heading,
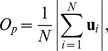
where 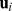 is the unit direction of fish number *i*. *O_p_* takes values between 0 (no alignment on average) and 1 (all fish are aligned). Second, the rotation order parameter *O_r_*, which describes a group's degree of rotation about its center of mass. To define this measure we introduce the unit vector 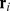 pointing from the shoal's center of mass towards fish *i*. The rotation order parameter *O_r_* is then defined by the mean (normalized) angular momentum
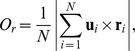
which, by construction also takes values between 0 (no rotation) and 1 (strong rotation).

Time series of these two order parameters give us valuable information about the global structure of a group and how the structure changes during an experiment. However, important pieces of information are not captured, like the group density, the average individual speed, and how close a group swims to the tank boundary. For a fuller picture of the collective dynamics, we will also use these order parameters. Other group properties could also be measured.

### Collective states exhibited and the role of group size

**Figure 1:**
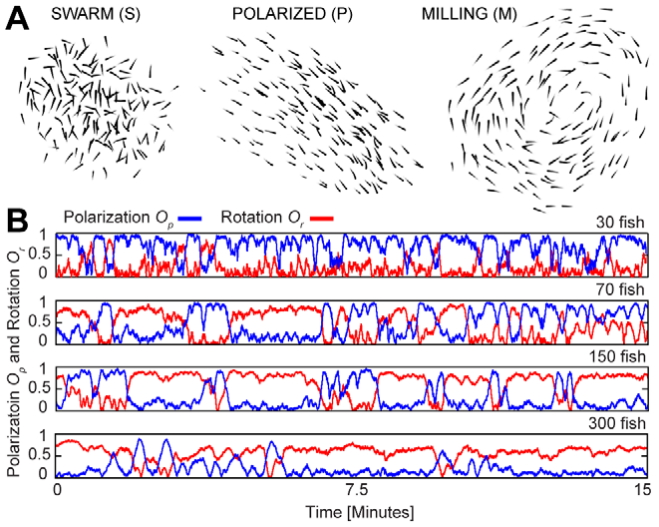
Dynamical states of schooling fish. (*A*) Snapshots of a group of 150 golden shiners swimming in a shallow tank. The different images (thresholded for clarity) demonstrate the typical configurations displayed by the fish school: swarm state (S), polarized state (P) and milling state (M). (*B*) Extracts of time series of order parameters for groups of 30, 70, 150, and 300 golden shiners. Polarization *O_p_* (in blue) measures how aligned the fish are, while rotation *O_r_* (in red) measures the degree of rotation around the center of mass of the fish shoal.

Throughout our entire period of filming the fish were cohesive, and for all group sizes three dynamically-stable collective behaviors were observed [3]; the *swarm* (S), *polarized* (P) and *milling* (M) group state. Snapshots of these distinct patterns are shown in Fig. 1A for a group of 150 fish (Videos S1, S2, S3, S4 contain video extracts of all group sizes). As in [3], these separate modes of motion can be categorized by only two structural properties (order parameters) of the group—its polarization *O_p_* and its degree of collective rotation *O_r_*. Groups repeatedly transitioned between these collective states, as is evident in representative time series of the order parameters shown in Fig. 1B.

**Figure 2:**
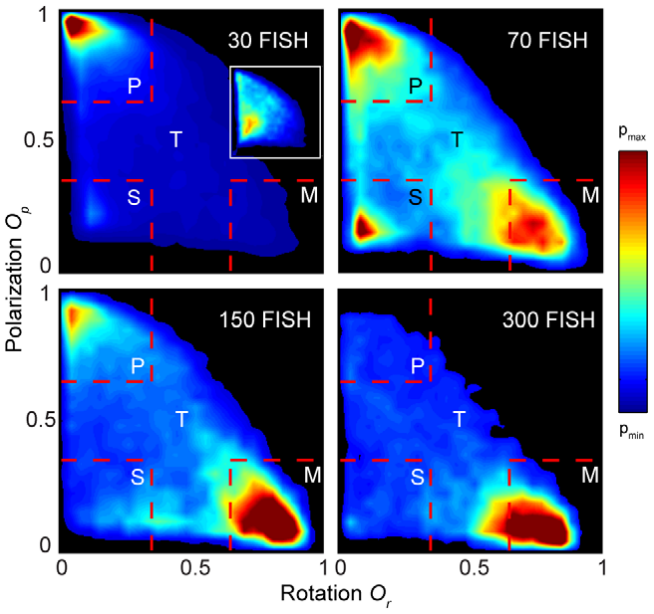
Density plots of polarization vs. rotation from experiments. The data shown are averaged over all replicates for each of the groups of 30, 70, 150, and 300 golden shiners. The order parameter space is divided into four regions—swarm (S), polarized (P), milling (M), and transition (T)—each being characterized by the dominant dynamical state of the fish school in that particular region. Different values of p_min_ and p_max_ were used for each group size to emphasize the density patterns and regions with no data are colored black. The insert in the 30 fish plot shows the density plot from an experiment with 30 fish and the tank area reduced to one tenth of the original.

To demonstrate more clearly these dynamically-stable states, in Fig. 2 we consider the proportion of time groups spend in different regions of the two-dimensional phase space spanned by the order parameters *O_p_* and *O_r_* (red representing more time spent in a given region and blue the least time; black areas signify regions of the phase space not visited by groups in our experiments). While all three states of motion were manifest in all groups, there are also visible differences relating to increasing group size. For the smallest group size of 30 fish we see that the polarized group state predominates (high *O_p_* and low *O_r_*). Only rarely did groups of this size exhibit swarm behavior (low *O_p_* and low *O_r_*), and even less frequently did they adopt the rotating group state (low *O_p_* and high *O_r_*). The fluctuations in the order parameters are also most frequent for this group size (Fig. 1B). For a group size of 70 fish the frequency of transitions decreases and the collective states corresponding to the three dynamically-stable states become clearly distinguishable as ‘hotspots’: the polarized state is no longer dominant, with milling and swarm behavior also being common. As group size is increased further, to 150 and then 300 fish, groups spend most of their time milling, displaying fewer transitions into (and among) the polar state and swarm state. For all group sizes the milling state has an equal probability of rotating clockwise and counter-clockwise, i.e. groups did not exhibit a handedness (see Fig. S1).

A natural consequence of increasing the number of fish in the tank is that the mean density within the experimental arena becomes higher, and hence the effects of the tank boundary become more pronounced. To reveal whether the higher density of fish per tank area in larger groups could cause the increased stability of the milling state we performed an experiment (4 replicates) with 30 fish in a smaller tank (0.66×0.38 m), which corresponds to the mean density of 300 fish in the larger tank. The density plot of the order parameters is shown as an inset in the 30 fish density plot in Fig. 2, and reveals that confinement by the boundaries and higher mean density do not lead to increased time spent milling. However, the time spent in the polarized state was reduced; contact with the smaller tank caused this group size to exhibit more frequent transitions to the swarm state than when it was in the larger tank. At least for the 30 fish, and possibly as a general result, the presence of the boundary does not increase the stability of the milling state per se. Rather, the stability of milling is largely determined by the size of the group. Although the functional reason for milling is not yet known, it does, however, allow individuals to be locally polarized, which could be important for information transfer, while allowing the group to remain in a specific area. Swarm behavior allows the group to remain in an area but is locally disordered and this may be more susceptible to predation [1].

**Figure 3:**
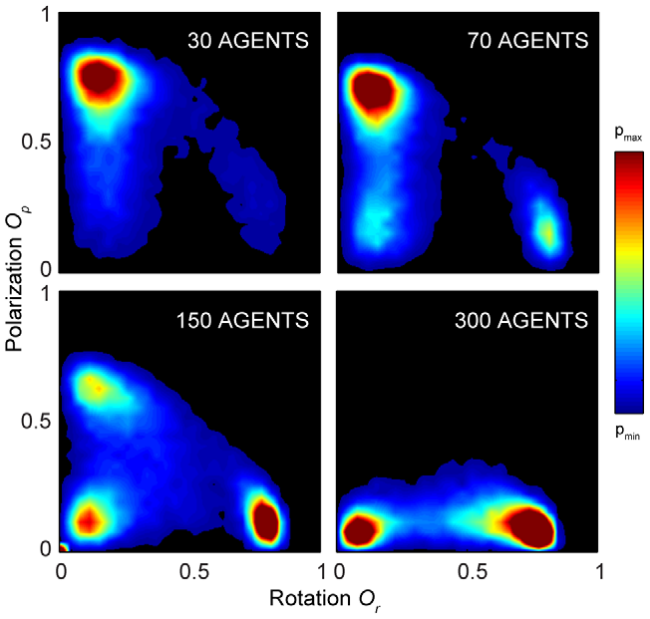
Density plots of polarization vs. rotation from simulations. The data shown are from simulations with 30, 70, 150, and 300 agents, employing a constant-speed agent based simulation model of collective behavior where no boundary is present (See Methods for simulation details). Regions with no data are colored black. As for the experimental data, the milling state grows in stability with group size.

To gain further understanding of the relationship between group size and the stability of the different group structures we employed the canonical model of grouping of Couzin et al. [3], in which there are no boundary interactions. Exploring the collective behavior of simulated individuals (see Methods for simulation set up and details) we find that the model produces qualitatively similar results across the range of group size in our experiments (30, 70, 150 and 300 agents), with the polarized states being dominant for the smallest group size, and an increasing proportion of the group's time is spent in the milling state as group size increases (see Fig. 3).

Another aspect of group size is the self-regulation of density. Theoretically, when more members are added to a group of self-propelled particles, the density can either remain approximately constant, in which case the system is H-stable, or the density can increase, and the system is catastrophic [4]. In our experiments, the individuals within the group regulate their spacing such that density tends to remain stable regardless of group size. The mean area occupied by the fish grows approximately linearly with group size and the packing fraction (and density) remains nearly constant (See Fig. S2). This regulatory behavior places the fish in the category of H-stable systems.

### Transitions between collective states

To quantify the relation between group size and collective state we need to explicitly define the different states. Given the relatively clear demarcation of states revealed by our data in Fig. 2 we employed a simple approach in which we discretize the phase space in terms of the order parameters. The specific range of values were motivated by the high-density regions observed in Fig. 2. We thus define that the school is in: the polar state (P) when O_p_>0.65 and O_r_<0.35; the milling state (M) when O_p_<0.35 and O_r_>0.65; and the swarm state (S) when O_p_<0.35 and O_r_<0.35. Outside these ranges we define the system to be in a transitional regime (T). On average, therefore, each region is characterized by the dominant dynamical state of the fish school within that particular region. The regions defining the dynamical states are overlaid the density plots in Fig. 2 (the qualitative nature of our results does not depend on the precise nature of how these regions are defined, see Fig. S3).

As shown in Fig. 1B, groups frequently transitioned between the three collective states. A transition is considered completed if the group moves from one of the three states to another. By this definition, a school can move from one of the dynamical states, into the transition region, and then back to its previous state, without having undergone a transition. We quantify, statistically, the transitions between states and investigate how the transition patterns depend on the group size.

**Figure 4:**
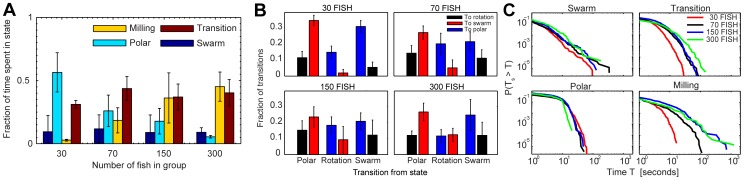
Statistics of state transitions. (*A*) Fraction of time spent in the different dynamical states shown for each group size. The error bars are showing the standard deviation measured across replicates. The group of 30 fish is predominantly in the polarized state, but less time is spent in this state with increasing group size (GLM: F1,22 = 36.21, P = 4.6e-06). As the group size increases, the groups gradually spend more time in the milling state (GLM: F1,22 = 19.31, P = 0.00023). The amount of time spent in the transition regime is high, but constant, for all group sizes (GLM: F1,22 = 0.67, P = 0.42). Across all group sizes (GLM: F1,22 = 0.053, P = 0.82) little time is spent in the swarm state. (*B*) Fraction of transitions from one state to another for the different groups. The error bars are showing the standard deviation measured across replicates. For all group sizes, the polar state predominantly transitions into the swarm state compared to the milling state (GLMM: F1,23 = 58.77, P<0.0001). The swarm state is dominated by transitions into the polarized state (GLMM: F1,23 = 55.69, P<0.0001). Although these transitions are consistent across group sizes, there is a significant interaction between group size and the frequencies of transitioning from the milling state to the swarm and polarized states (GLMM: F1,22 = 13.30, P = 0.0014). While the milling state tended to transition into the polar state, for 300 fish there was a roughly equal probability of transitioning to the polar or swarm states. (*C*) Rank plots showing the probability of being in a state longer than time T_s_ before moving into a different regime. There was no significant difference between group sizes in the persistence of the polar state (GLMM: F1,22 = 0.58, P = 0.45), although group size increased the persistence of the transition (F1,22 = 24.52, P = 1e-04) and milling states (F1,22 = 17.54, P = 4.0e-04), and to a lesser degree, the swarm state (F1,22 = 5.31, P = 0.031).

As we previously saw in Fig. 2, the proportion of time spent in the polarized state decreases strongly with group size from 0.57 to 0.26, 0.18 and 0.05 (30 to 300 fish shoals, respectively, light blue columns in Fig. 4A). Likewise, the proportion of time in the milling state increases with group size from 0.03 to 0.18, 0.36 and 0.45 (yellow columns Fig. 4A). The group size has however little effect on the proportion of time spent in the swarm state or in the transition region, which varies between 0.09 and 0.11 (dark blue columns Fig. 4A), and 0.31 and 0.44 (brown columns in Fig. 4A), respectively. The fraction of transitions between states, as illustrated by Fig. 4B, exhibit little variation between the group sizes. The only visible trend is a small increase in the number of transitions between the milling state and the swarm state (see also Fig. S4 for alternative graphics).

To complement this picture it is important to note two (inter-related) features that do change with group size: Firstly the rate at which groups exhibit transitions decreases as a function of group size (from 2.0 transitions/min for 30 fish, to 1.4, 1.2, and 0.8 transitions/min for 70, 150, and 300 fish respectively); Secondly, the stability of the milling state increases as a function of group size (the longest time a group of 30 spent milling was 17 s, this increased to 110 s, 708 s, and 1245 s for group sizes 70, 150 and 300, respectively. See Fig. 4C for rank plots of time spent in a state before transitioning). This means that the large proportion of time the 30 fish spent in the polarized state is an accumulated effect of many visits into the state, while the proportion of time the 300 fish spent in the milling state is greatly affected by the milling state being more stable for larger groups.

A further feature of the transitional behavior of groups is that transitions from one dynamical state to another only accounted for 47% (n = 1943) of the total number of visits into the transition zone (n = 4119), counting only visits lasting longer than 1 s. This demonstrates that the schools experience frequent perturbations, of which some result in transitions to another state (Fig. 4B), and the others back to the preceding state.

From observing the schooling behavior it appears that perturbations to the group act as triggers to transitions between collective states (see Videos S1, S2, S3, S4) and we can identify two main sources for fluctuations that result in transitions; interactions with the tank wall (boundary effects) and fluctuations due to the intrinsically noisy nature of individual motion. We note that these processes are not mutually exclusive.

### Boundary effects and state transitions

**Figure 5:**
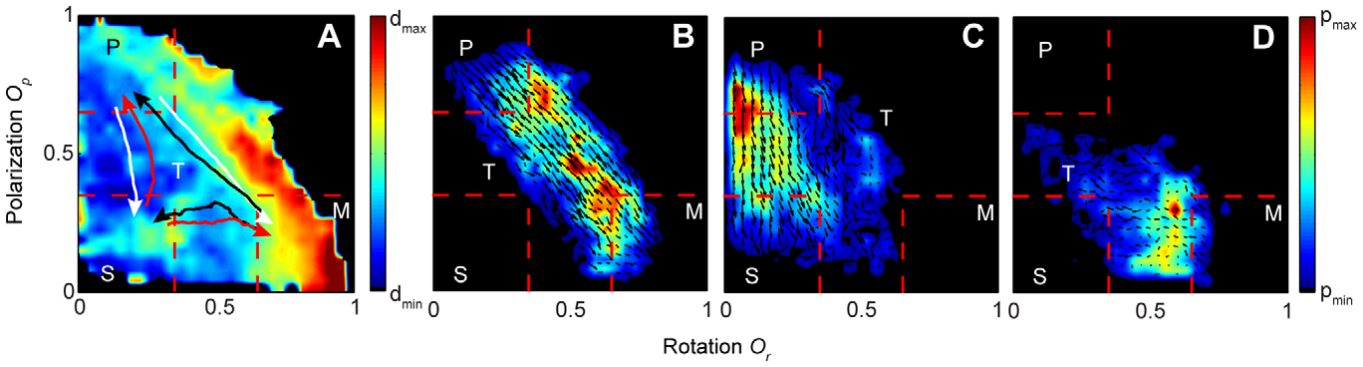
Transition patterns for 150 fish. (*A*) Density plot of the smallest distance from the center of mass of the fish shoal to the tank boundary as a function of rotation and polarization (d_min_ = 26 and d_max_ = 52 cm). The overlaid arrows are the averaged trajectories of all transitions in the rotation-polarization phase space. (*B–D*) Density plots of transitions from polarized to milling state (*B*), from polarized to swarm state (C) and from milling to swarm state (D). Overlaid the density plots are the corresponding velocity fields of the transition data (in the rotation-polarization phase space). Plots of the reverse transitions and group sizes 30, 70 and 300 are provided in SI.

In order to reveal more clearly the role of boundary effects on state transitions we use our extensive time series to characterize the typical nature of transitions and relate these to whether the group tends to be relatively close to, or far from, the boundary. We present data for 150 fish in Fig. 5 (for other group sizes see Fig. S5). In Fig. 5A the arrows represent the average trajectories that groups take through *O_p_* and *O_r_* space when transitions occur and, unlike Fig. 2, the density plot now depicts the distance *d_b_* from the center of mass of the group to the closest point at the tank boundary; red colors represent a relatively large distance and blue colors relative proximity to the boundary. A more detailed view of the transition dynamics is presented in Figs. 5B–D. Here, for each of the transitions from polar to milling, polar to swarm and milling to swarm state, the average trajectories are plotted as a velocity field in the *O_p_* and *O_r_* phase space overlaid on the density plot showing the distribution of trajectories (for the reverse transitions and other group sizes see Figs. S6 and S7).

The data shown in Fig. 5 verify that transitions happen both close to the wall of the tank, and in the center of the tank. For large parts of the transition region schools are relatively close to the boundary, such as from the polarized state to the swarm state, where most transitions take place (Fig. 5C). Although not as clear, the transitions between the milling state and the swarm state are also characterized by being, on average, closer to the wall (Fig. 5D). The exception is for transitions that occur with high values of the order parameters, that is, between the polarized state and the milling state (Fig. 5B), and vice versa. These tend to occur both close to and also away from the wall. This indicates, as evidenced by the video footage (Videos S1, S2, S3, S4), that both boundary and other triggering mechanisms are important for inducing transitions between collective states.

**Figure 6:**
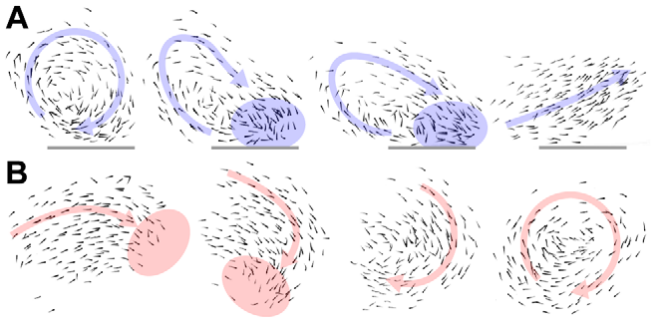
Time-lapse examples demonstrating transition mechanisms. (*A*) Transition initiated by interaction with the tank wall. In the first picture, the fish is in a milling state, indicated by the blue arrow, and the lower part of the group is close to the tank wall (grey line). In the second picture, the interaction with the wall has caused a local increase in density, marked by the blue region, and a few individuals have started to turn opposite the milling direction. This cascades, and in the third picture the flow of the mill is interrupted as a large proportion is breaking away from the milling direction. The result is total unraveling of the milling state and transition into the polar state, seen in the last picture. (*B*) Transition from the polar state to the milling state, initiated by individuals in the shoal. In the first picture, the group is in a polar state, signified by the red arrow. A few individuals in the front, visible in the red region, have started to turn downwards. This leads the group as a whole into a sharp right turn, and as picture two demonstrates, the group is forced into a shape with larger curvature. Now, when the individuals in front of the group can spot the back of the group, they continue the turning and start following the back, as seen in the third picture. In the final picture, as the front individuals catches up with the tail, the loop closes and the transition into the milling state is complete.

When in the milling state, interactions with the boundary can result in a local increase in density near the wall, due to the inherently constrained nature of motion when abutting the boundary. This can cause the mill to transition into a polarized state as shown in Fig. 6A. Another way in which the mill can break down is due to the action of individuals at the group edge; if fish turn or move away from the edge of the group this can seed the unraveling of the milling formation into a polarized state. Conversely, when in a polarized state individuals at the front of the group can turn towards the main mass resulting in a perturbation that prompts the group to turn, potentially initiating the mill formation. This last example is evidenced in Fig. 6B and demonstrates that the milling formation can emerge as a group effect from the individual interactions—without direct interaction with the tank boundary.

In the case of polarized groups the boundary also has inevitable consequences on transitions; a polarized group may swim directly towards a wall, or corner, of the tank. Individuals reaching the wall slow down and tend to become disordered (unaligned) and the group can transition into the swarm state. That this mechanism of transition is dominating is demonstrated both by the average transition path from polarized to swarm in Fig. 5A, which crosses a region where *d_b_* is small, as well as the short average time groups spend in the polarized state before transitioning, as shown in Fig. 4C.

### The relationship between speed, density and group state

For tractability many previous models of animal grouping (including that of Couzin et al. [3]) have assumed that individuals move at constant speed and social response is represented by adjusting direction of travel in response to the positions and/or orientations of near neighbors. Recently, however, two experimental studies on schooling fish, Katz et al. [17], involving golden shiners (*Notemigonus crysoleucas*—the species used here), and Herbert-read et al. [18], involving mosquitofish (*Gambusia holbrooki*), have highlighted the importance of speed regulation to collective behaviors. In the former study it was found that individual social interactions can be approximated qualitatively by pairwise interactions that are functions of the position and speed of each individual. While the spatial nature of these interactions was found to be relatively independent of individual speed, the magnitude, or strength, of response to neighbors decreased greatly as individual speed decreased. Also Viscido et al. [24] found a positive correlation between average group speed and polarity for shoals of 4 and 8 giant danios (*Devario aequipinnatus*). This suggests that there is an important relationship between individual speed and the degree to which individuals coordinate their motion with neighbors, a relationship that is not captured in many models of collective motion [3], [15], [25], [26].

**Figure 7:**
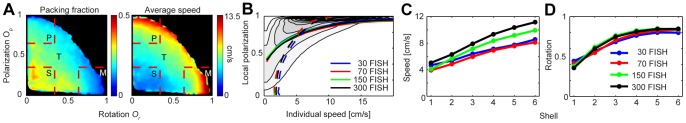
Structural properties. (*A*) Density plots of packing fraction and average individual speed (averaged per frame) as functions of rotation *O_p_* and polarization *O_r_* for 150 fish. (*B*) The plot illustrates the correlation between individual speed and local polarization estimated in two ways from the underlying density maps (the example shown in the background is for 150 fish). The stapled curves are produced by averaging across individual speeds for each value of the order parameter; the solid curves from averaging across the order parameter values for each individual speed. The local polarization of an individual fish is defined as the polarization Op restricted to the area inside a circle with radius 15.6 cm (approximately 3 body lengths) centered at the individual fish. (*C*) Average individual speed at different radial positions in the milling state. (*D*) Average rotational order parameter at the same positions. The radial division of the milling state in (*C*) and (*D*) is constructed by centering six shells outside each other, where the outermost shell has a radius defined by the distance from the group's center of mass to the median distance of the five most peripheral fish (see illustration in Fig. S11). The width of each shell is the radius of the outer shell divided by six. The averages are calculated for each shell, where the outer shell even includes peripheral fish.

Examining the relationship between the mean speed of individuals in the group and the ‘packing fraction’ (a measure of the density of individuals within the group) and the order parameters *O_p_* and *O_r_*, we observe that low speed is associated with the group being relatively dense and both locally- and globally-disordered (the swarm state). The two locally-ordered (milling and polarized) states are characterized by higher mean speed and a decreased packing fraction (see Fig. 7A for group size of 150 fish; this relationship is common among all group sizes as shown in Fig. S8). Consequently the relationship between density and order is the *opposite* of that predicted by the most studied models of grouping behavior, notably the Vicsek model [15]; although we note that such simple models have been extremely useful in developing understanding of group dynamics for other animal aggregates, such as locusts [14], and other species of schooling fish [23].

From our data we cannot distinguish between two, not mutually exclusive, hypotheses regarding the causal relationship between speed and order; does decreasing speed induce local disorder through weakened social interactions, or does perception of local disorder reduce an individual's speed? Since golden shiners do not appear to explicitly respond to the body orientation of neighbors, and rather respond more-or-less exclusively to individuals' positions in space [17], increasing speed likely increases local order. However, a dense, slow moving and disordered group is also likely to further reduce individual speed (not least through increased risk of collision) - thus both causal relationships likely co-exist.

**Figure 8:**
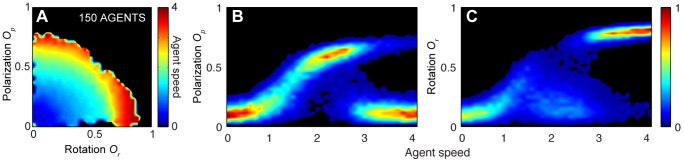
Relationship between agent speed and order in constant-speed agent based simulation model with 150 agents. (*A*) Density plot of agent speed as function of rotation *O_r_* and polarization *O_p_*, revealing a bistable regime between the milling and the polar states for high speeds. (*B*) Normalized probability plot of polarization *O_p_* as function of agent speed. (*C*) Normalized probability plot of rotation *O_r_* as function of agent speed. The last two plots illustrate the bifurcation that occurs as the speed is increased, where the system transitions from a swarm state and is found either in a highly polarized state or in a milling state. (See Methods for simulation details).

To demonstrate the plausibility (and indeed, generality) of speed-induced transitions, we return to the model employed above, from [3]. We verify that changing individual speed does result in qualitatively the same transitional behavior seen here; swarm behavior for relatively low speed and bistable milling and parallel group motion as individual speed increases. This result holds up to 150 agents. For groups of 300 agents the milling state is dominant and no instances of the polar state are found. (see Fig. 8 for results from simulations with 150 agents and Fig. S9 for remaining group sizes. Simulation details are found in Methods).

### Local social interactions are similar across group size and dynamical state

In order to deepen our understanding of the local dynamics we also quantified the relationship between the speed of an individual and the degree of order in its immediate vicinity (within a radius distance of 15.5 cm). In Fig. 7B we show the resulting relationship as a contour plot for groups of 150 fish (see Fig. S10 for corresponding plots of 30, 70 and 300 fish, and for different radial proximity distances). A strong association is evident between individual speed and local order. Assuming an unknown causal direction in the relation between the speed and the local order, as discussed above, there are two ways we can average the contour plots; either over all values of the speed for each value of the local order, or vice versa, for each value of the speed we average over the local order. The results of both procedures, for all group sizes, are overlaid on the contour plot in Fig. 7B. While the two approaches yield disparate curves, they both demonstrate a similar relationship between individual speed and local order. Although the fish have to align at higher speeds to maintain group cohesion, it is unclear why they should become disordered at low speeds. There is also a much greater variance in local order at low speeds, demonstrating a wide degree of flexibility when individual speeds are low. Interestingly, across the group sizes the two sets of curves are close to identical. This suggests that, from the perspective of a focal individual, it may simply adopt the same local rules regardless of group size (consistent with the findings of Katz et al. [17] for groups of 10 and 30 fish).

Contrary to the polarization order parameter, the rotation order parameter has little meaning on a purely local scale. Rather we compute the rotation order parameter separately for a series of shells placed around the center of mass of the group, as illustrated in Fig. S11. This allows us to obtain a well-defined measure that provides insight into the structural organization of the milling state. As can be seen in Figs. 7C and 7D this state is characterized by a center with low speed and low degree of structure that contributes little to the milling state, while as we move towards the edge of the group the speed and impact from each shell on the milling state increases. In Fig. 7D the curves are almost identical for all group sizes suggesting that, again, scaling the size of the group has little effect on the local structural signature of this collective state.

These results demonstrate that the ordered polarized and milling states are locally near identical from the perspective of a focal individual, regardless of group size. These data also support the prediction of a multi-stable locally-ordered regime in which the group can transition back and forth between the polarized and milling state through stochastic and boundary-induced effects.

### Conclusions

Despite the multitude of local interactions that result in coordinated group motion we demonstrate that schooling golden shiners predominantly exist in three ‘fundamental’ dynamically-stable states of the underlying dynamics: swarm, milling and polarized motion. We establish that group states, and transitional behavior, can be represented in low-dimensional space, a projection that allows us to see the path taken by groups between the three dynamically-stable states as well as to relate the collective states exhibited to properties such as group size, individual speed and perturbations to the group. We note that it is possible that further collective states may be found within the classified dynamically stable regimes described here, but the present states are highly consistent with the theoretical predictions of three regimes

A key question in the study of collective behavior is whether different group-level patterns result from all individuals responding to the same external factor [27], or individuals changing behavior [1], or whether multiple dynamically-stable collective states co-exist for the same individual behaviors [3]. Our results provide evidence for the importance of the two latter processes in the behavior of schooling fish: transitions from the swarm, to the milling or parallel group states (and vice versa) involve a social feedback whereby individuals adjust behavior—in this case their speed—in response to prevailing local conditions. Low average speeds among group members correspond to them occupying the dense, disordered swarm regime.

Higher speeds correspond to higher local order (alignment among group members) and groups existing in either the milling or polarized state. Transitions between these states occur with negligible, or no, change in local density, order or speed; instead perturbations such as collisions with the boundary, or (seemingly stochastic) fluctuations in motion at the group edge (in the case of milling to polarized state transitions) or front (in the case of polarized to milling transitions) result in the group leaving one dynamically-stable state, and either then returning to that state, or transitioning to the alternative locally-ordered regime. Thus the milling and polarized states appear to be bistable; the state exhibited by the group effectively being dependent on starting conditions and/or the nature of perturbations, as well as the group size. Theoretically [3] and experimentally (analysis of shoals of 30 fish in small tank), milling states are seen to be less stable for small groups, when controlling for boundary condition effects. It is likely that the relationship we found between speed and local order is a generic feature of mobile groups with local interactions. Furthermore, qualitatively similar features have been observed in small groups (4 and 8 fish) of the giant danios [24].

A key challenge for animal behavior in this, and future, decades is to understand how the microscopic mechanisms of interactions among molecules, physiological systems and neural circuits result in behavior at higher levels of organization. Whereas we focused on collective behavior resulting from interactions among individual organisms, the general approach adopted shares commonalities with approaches that have successfully been used to characterize the dynamical properties of gene interaction networks [28], neuronal circuits [29], how locomotion is coordinated among limbs, each of which has many degrees of freedom [30], and how the behavior of individual organisms (such as *Caenorhabditis elegans*), despite apparent complexity, can be deconstructed into a discrete number of low-dimensional behavioral dynamically-stable states (see Stephens et al. [31]). We suggest that development, and adoption of, such techniques in the behavioral sciences could facilitate the advent of increasingly integrative and quantitative insights.

Our work demonstrates that such an approach to data collection and analysis can reveal underlying simplicity in the dynamical properties of collective behavior in groups. Collective behavioral states appear to result from both behavioral feedback processes whereby individuals both adopt, and influence, the behavior of near neighbors and also as multi-stable regimes in which individual behavior does not change, but rather perturbations induce relatively abrupt transitions between alternate and co-existing dynamically-stable behavioral states. Prey groups have been observed to switch states upon detecting a predator [32] and risk can be dependent on these states [9], [33]. Whether the mechanisms for switching between states as identified here are somehow themselves adaptive would be an interesting question to address in future work.

## Methods

### Experiments and data extraction

The experimental setup, the automated tracking procedure and the methods for constructing detailed trajectory data were the same as described in Yael et al. [17] and details are found there. The data used for the analyses in this work are time series of positions and velocities of the individual fish. The tracking accuracy varied with the structure of the groups. As we show in Fig. S12, ordered groups were more precisely tracked, while dense and disordered groups were more prone to tracking errors. On average the percentage of frames with tracking accuracy above 80% were 88% for 30 fish, 91% for 70 fish, 80% for 150 fish and 71% for 300 fish. Since our focus in this paper does not rely on us maintaining identities for long periods of time there is ample data from which to calculate global patterns and distributions of properties such as speed.

### Order parameters

We calculated the polarization order parameter *O_p_* and the rotation order parameter *O_r_* (see definitions in Results and Discussion) for each frame and smoothed the resulting time series using a moving average with a span of 30 frames (corresponding to 1 second). In Fig. 7B we used a definition of the polarization order parameter that is restricted to the local neighborhood around a focal individual. Similarly, we used a radial version of the rotation order parameter in Fig. 7D, in which only fish inside a shell of given radius surrounding the full shoal's center of mass were included.

### Density plots

All *O_r_-O_p_* density plots were made by dividing up the phase space into 30 times 30 bins and counting the number of values falling into the respective bins (Figs. 2, 3 and 5B–C) or calculating the average value in the bins (Figs. 5A and 7A). Only bins with counts above a certain threshold were included (100 counts in Figs. 2 and 7A and 20 counts in Fig. 5). Before plotting, the bin values were interpolated over a finer mesh of 300 times 300 points.

### Defining states

Deciding exact thresholds for when a shoal is in a certain state or not is hardly possible, even though it is easy to approximately mark out the regions in the O_r_-*O_p_* phase space that corresponds to the swarming, polar and milling states. Since the analyses we did were not critically dependent on whether precise demarcations could be made, we used a heuristic approach and—motivated by the high density regions observed in Fig. 2, as well as visual verification from the videos—defined the dynamic states as follows: polar state (P) when *O_p_*>0.65 and *O_r_*<0.35; milling state (M) when *O_p_*<0.35 and *O_r_*>0.65; and swarm state (S) when *O_p_*<0.35 and *O_r_*<0.35. Outside these ranges we defined the system to be in a transitional state (T).

### Packing fraction

To calculate the packing fraction of a group we first used an alpha-shape algorithm [34] to measure the area spanned by the group. Dividing the number of fish by the measured area and then multiplying by the average area of a fish body (40 times 5 pixels) produced the packing fraction value.

### Transition paths

To calculate the average transition paths in Fig. 5A we first interpolated all transition time series to have the same length. Then we averaged the interpolated transition paths between one state and another. The vector plots in FigS. 5B–D were constructed by first differentiating each of the transition time series to create a velocity vector field, which we then coarse-grained by dividing the *O_r_-O_p_* phase space into 30 times 30 bins and averaging over the vector field in each bin.

### Simulations

Simulations were performed using the constant-speed agent-based model described in Couzin et al. [3] with 30, 70, 150 and 300 individuals. In this model the individuals move with constant speed and interact with each other through three types of interactions: repulsion, alignment of orientation and attraction. Centered on each individual are three spherical non-overlapping behavioral zones; zone of repulsion, zone of orientation and zone of attraction. The distribution of neighbors across these three zones is what decides the desired heading of an individual. For a detailed description of the model algorithm, see [3]. In our simulations we varied the speed from 0.1 to 4.1 unit lengths per unit time in increments of 0.1 and performed 500 replicates for each value of the speed. Each simulation was run for 2500 time steps and the order parameters in the final simulation step recorded. The remaining model parameters remained fixed throughout the simulations and were: zone of repulsion 1; zone of orientation 3; zone of attraction 15; field of perception 270 degrees; turning rate 60 degrees; error 0.2 radians; time step increment 0.1. Note that the simulations are not parametrized to fit the schools of fish. Rather, we use the simulations to display a generic quality of self-propelled particle models that aligns with experimental observations.

### Statistical analyses

The frequency of the milling state rotating in a clockwise or counter-clockwise direction was analysed using a quasi-poisson distributed Generalised Linear Mixed Model (GLMM). Direction (a within-subject fixed factor) and group size (between-subject covariate) were the explanatory variables along with their interaction term, and shoal identity the random variable. The time spent in each state as a proportion of the total trial time was calculated for each group of fish, and then was analyzed as a function of group size using quasi-binomial Generalised Linear Models (GLM). Each state was analyzed separately, as well as the time spent in transition between states. Quasi-negative binomial GLMMs were used to analyse the frequency of transitions from each state (swarm, milling and polarised) to a different state (the ‘to’ state, a within-subject fixed factor), again with group size as an additional explanatory variable and shoal identity the random variable. These models were run separately for each ‘from’ state and included the ‘to’ state×group size interaction. The persistence of each visit to a state before transitioning into a different state (i.e. its stability) was analysed using quasi-negative binomial GLMMs. As a visit to a state within one shoal was not independent from the duration of other visits within that shoal, the analysis was carried out separately for each state. Group size was used as an explanatory variable and shoal identity as the random variable. The parameter theta for the quasi-negative binomial GLMMs was estimated from running negative-binomial GLMs without the random variable first, which gives an estimate for theta. Non-significant interaction terms were removed before testing main effects, but with the statistics given for main effects including any other main effects regardless of their significance. All statistical tests were carried out using R 2.14.2.

## Supporting Information

Figure S1 **No signature of handedness.** The plot shows the mean and standard deviation of the number of transitions (per replicate) resulting in a clockwise (blue) or counter-clockwise (red) milling state. Transitions into the milling state were no more likely to go clockwise or counterclockwise (GLMM: F1,23 = 0.7191, P = 0.4052), and neither was this affected by group size (direction×group size interaction: F1,22 = 2.9966, P = 0.0974).
(TIF)

Figure S2 **Relation between group size and group area and packing fraction.** (*A*) shows the mean group area plotted as a function of group size, including standard deviations. The dashed red line is a linear fit. (*B*) shows the mean packing fraction as a function of group size, also with standard deviations incuded.
(TIF)

Figure S3 **Statistics of state transitions for varying definitions of dynamical state.** We define the dynamical states as: polar state (P) when O_p_>1−k and O_r_<k; milling state (M) when O_p_<k and O_r_>1−k; and swarm state (S) when O_p_<k and O_r_<k. The plots show the transition statistics for k = 0.25 (first row), 0.35 (second row and the value used in the paper—included for ease of comparison) and 0.40 (third row). As in the paper: (A) Fraction of time spent in the different dynamical states shown for each group size. The error bars are showing the standard deviation measured across replicates. (B) Fraction of transitions from one state to another for the different groups.
(TIF)

Figure S4 **Schematic overview of transitions between dynamical states.** The filled circles represent the fraction of time spent in a dynamical state and the arrows represent the fraction of transitions from one dynamical state to another. The absolute number of transitions from one state to another is placed at the tip of the respective transition arrow.
(TIF)

Figure S5 **Average transition paths.** Density plot of the smallest distance from the center of mass of the fish shoal to the tank boundary as a function of rotation and polarization. The overlaid arrows are the averaged trajectories of all transitions in the rotation-polarization phase space. For the different group sizes we used d_min_ = 19.5 cm and d_max_ = 36 cm (30 fish), d_min_ = 23 cm and d_max_ = 49 cm (70 fish), d_min_ = 26 cm and d_max_ = 52 cm (150 fish), d_min_ = 29 cm and d_max_ = 55 cm (300 fish). Both the distance distribution and the transition paths are similar across group sizes. The transitions between the swarm state and the polarized state on average happen when the center of mass is close to the boundary, indicating that these transitions are mostly caused by interactions with the boundary. The same is the case with the transitions between the swarm state and the milling state, if not as clear. Between the polar state and the milling state, the transitions on average happen further away from the boundary, suggesting that these transitions can be caused both by interactions with the boundary or by local perturbations in the school.
(TIF)

Figure S6 **Transition patterns.** Density plots of transitions between states for (*A*) 30 fish and (*B*) 70 fish. Overlaid the density plots are the corresponding velocity fields of the transition data (in the rotation-polarization phase space).
(TIF)

Figure S7 **Transition patterns.** Density plots of transitions between states for (*A*) 150 fish and (*B*) 300 fish. Overlaid the density plots are the corresponding velocity fields of the transition data (in the rotation-polarization phase space).
(TIF)

Figure S8 **Packing fraction and average speed.** Density plots of packing fraction and average individual speed (averaged per frame) as functions of rotation *O_p_* and polarization *O_r_* for (*A*) 30 fish, (*B*) 70 fish and (C) 300 fish.
(TIF)

Figure S9 **Relationship between agent speed and order in constant-speed agent based simulation model with 30, 70 and 300 agents.** (*A*) Density plot of agent speed as function of rotation *O_r_* and polarization *O_p_*, revealing a bistable regime between the milling and the polar states for high speeds in simulations with 30 and 70 agents. For 300 agents the milling state becomes dominant and the bistable regime dissappears. (*B*) Normalized probability plot of polarization *O_p_* as function of agent speed. (*C*) Normalized probability plot of rotation *O_r_* as function of agent speed. (See Methods for simulation details).
(TIF)

Figure S10 **Relation between local polarization and individual speed.** The plots show the correlation between individual speed and local polarization estimated in two ways from the underlying density maps. The stapled curves are produced by averaging across individual speeds for each value of the order parameter; the solid curves from averaging across the order parameter values for each individual speed. The local polarization of an individual fish is defined as the polarization *O_p_* restricted to the area inside a circle centered at the individual fish. The plots show the results from using neighborhoods of 1–3 body lengths (BL), where BL is 5.2 cm.
(TIF)

Figure S11 **Radial division of milling state.** The figure shows the division of fish in the milling state into shells, where the distance of the outer shell (dashed lines) is defined as the median distance to the group's centre of mass of the five most peripheral fish, and the width of each shell is the radius of the outer shell divided by six.
(TIF)

Figure S12 **Tracking accuracy.** (*A*) shows histograms of how many fish are tracked in each frame. The red line in each histogram denotes the threshold for 80% tracking accuracy. The percentage of frames above 80% accuracy are 88% for 30 fish, 91% for 70 fish, 80% for 150 fish and 71% for 300 fish. (*B*) shows the density distributions of tracking accuracy as a function of the rotational (*O_r_*) and polarization (*O_p_*) order parameters.
(TIF)

Video S1 **30 golden shiners swimming in shallow water in the laboratory.** The video plays at 16× normal speed to clearly depict the different dynamical patterns occurring.
(M4V)

Video S2 **70 golden shiners swimming in shallow water in the laboratory.** The video plays at 16× normal speed to clearly depict the different dynamical patterns occurring.
(M4V)

Video S3 **150 golden shiners swimming in shallow water in the laboratory.** The video plays at 16× normal speed to clearly depict the different dynamical patterns occurring.
(M4V)

Video S4 **300 golden shiners swimming in shallow water in the laboratory.** The video plays at 16× normal speed to clearly depict the different dynamical patterns occurring.
(M4V)
