# Supplementary figures and images for: Collective States, Multistability and Transitional Behavior in Schooling Fish
Source: PLoS Comput Biol. 2013 Feb 28;9(2):e1002915. doi: 10.1371/journal.pcbi.1002915 (PMC3585391; doi:10.1371/journal.pcbi.1002915)

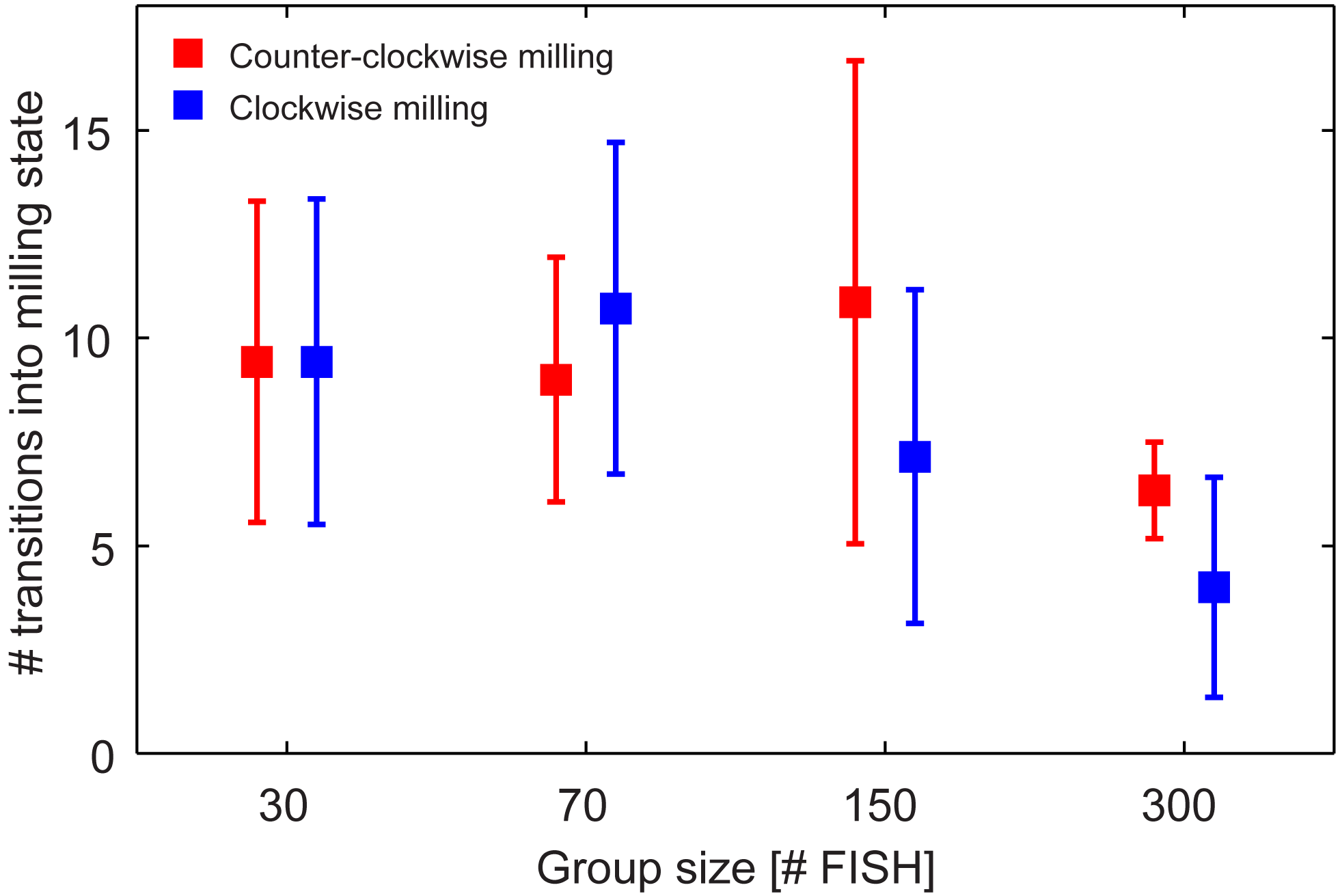

Supplement: Figure S1 — No signature of handedness. The plot shows the mean and standard deviation of the number of transitions (per replicate) resulting in a clockwise (blue) or counter-clockwise (red) milling state. Transitions into the milling state were no more likely to go clockwise or counterclockwise (GLMM: F1,23 = 0.7191, P = 0.4052), and neither was this affected by group size (direction×group size interaction: F1,22 = 2.9966, P = 0.0974). (TIF) [file pcbi.1002915.s001.tif]

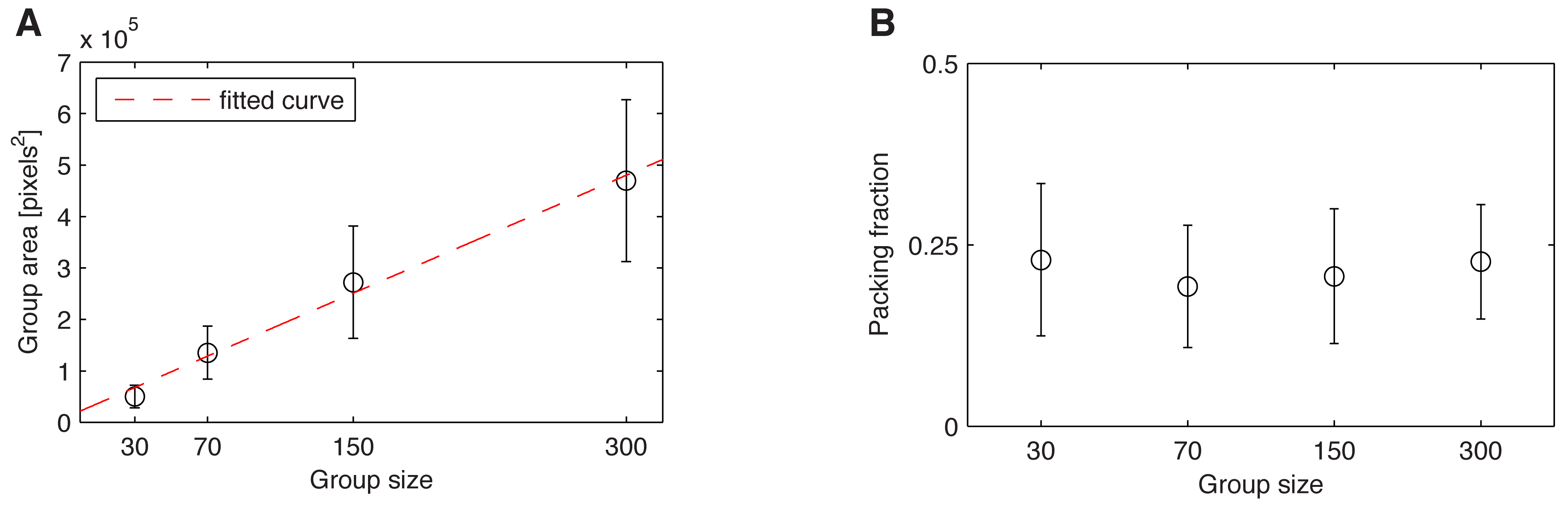

Supplement: Figure S2 — Relation between group size and group area and packing fraction. (A) shows the mean group area plotted as a function of group size, including standard deviations. The dashed red line is a linear fit. (B) shows the mean packing fraction as a function of group size, also with standard deviations incuded. (TIF) [file pcbi.1002915.s002.tif]

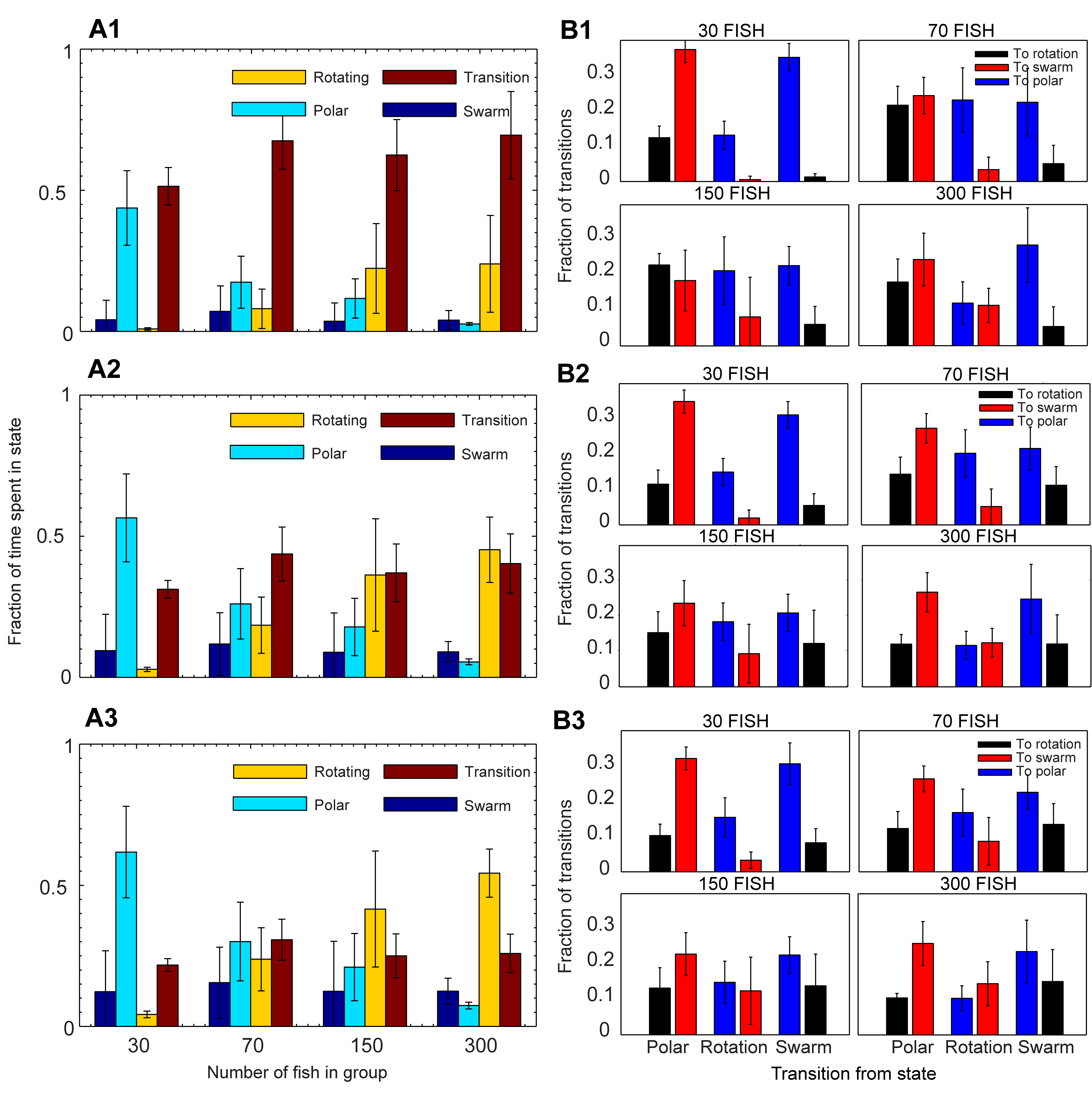

Supplement: Figure S3 — Statistics of state transitions for varying definitions of dynamical state. We define the dynamical states as: polar state (P) when Op>1−k and Or<k; milling state (M) when Op1−k; and swarm state (S) when Op<k and Or<k. The plots show the transition statistics for k = 0.25 (first row), 0.35 (second row and the value used in the paper—included for ease of comparison) and 0.40 (third row). As in the paper: (A) Fraction of time spent in the different dynamical states shown for each group size. The error bars are showing the standard deviation measured across replicates. (B) Fraction of transitions from one state to another for the different groups. (TIF) [file pcbi.1002915.s003.tif]

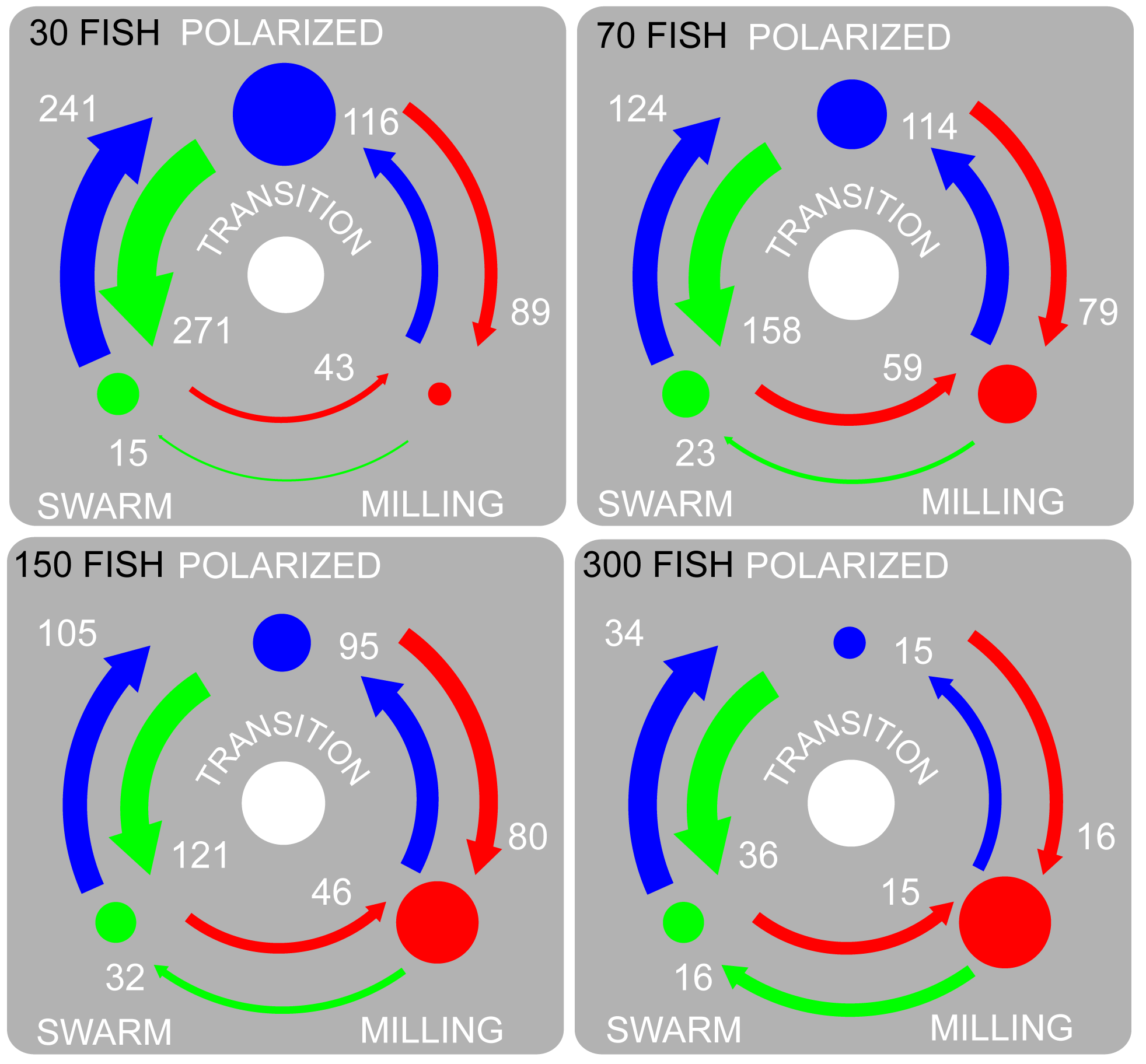

Supplement: Figure S4 — Schematic overview of transitions between dynamical states. The filled circles represent the fraction of time spent in a dynamical state and the arrows represent the fraction of transitions from one dynamical state to another. The absolute number of transitions from one state to another is placed at the tip of the respective transition arrow. (TIF) [file pcbi.1002915.s004.tif]

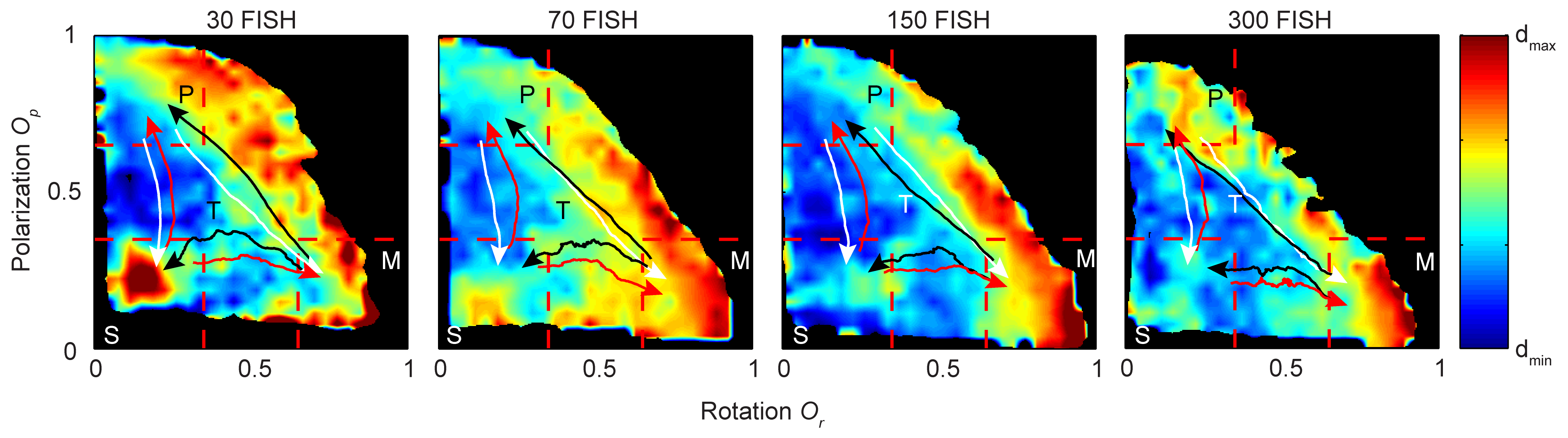

Supplement: Figure S5 — Average transition paths. Density plot of the smallest distance from the center of mass of the fish shoal to the tank boundary as a function of rotation and polarization. The overlaid arrows are the averaged trajectories of all transitions in the rotation-polarization phase space. For the different group sizes we used dmin = 19.5 cm and dmax = 36 cm (30 fish), dmin = 23 cm and dmax = 49 cm (70 fish), dmin = 26 cm and dmax = 52 cm (150 fish), dmin = 29 cm and dmax = 55 cm (300 fish). Both the distance distribution and the transition paths are similar across group sizes. The transitions between the swarm state and the polarized state on average happen when the center of mass is close to the boundary, indicating that these transitions are mostly caused by interactions with the boundary. The same is the case with the transitions between the swarm state and the milling state, if not as clear. Between the polar state and the milling state, the transitions on average happen further away from the boundary, suggesting that these transitions can be caused both by interactions with the boundary or by local perturbations in the school. (TIF) [file pcbi.1002915.s005.tif]

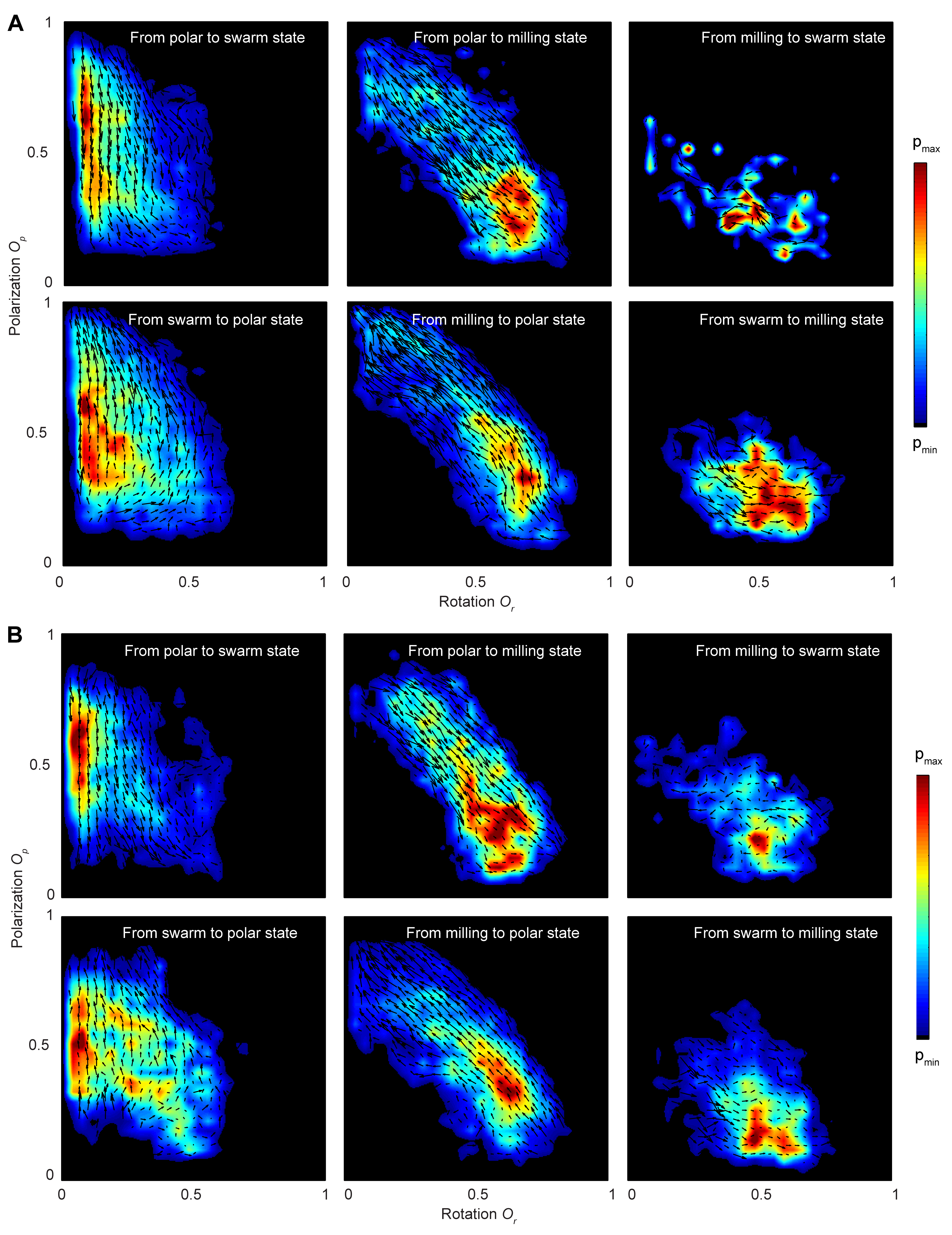

Supplement: Figure S6 — Transition patterns. Density plots of transitions between states for (A) 30 fish and (B) 70 fish. Overlaid the density plots are the corresponding velocity fields of the transition data (in the rotation-polarization phase space). (TIF) [file pcbi.1002915.s006.tif]

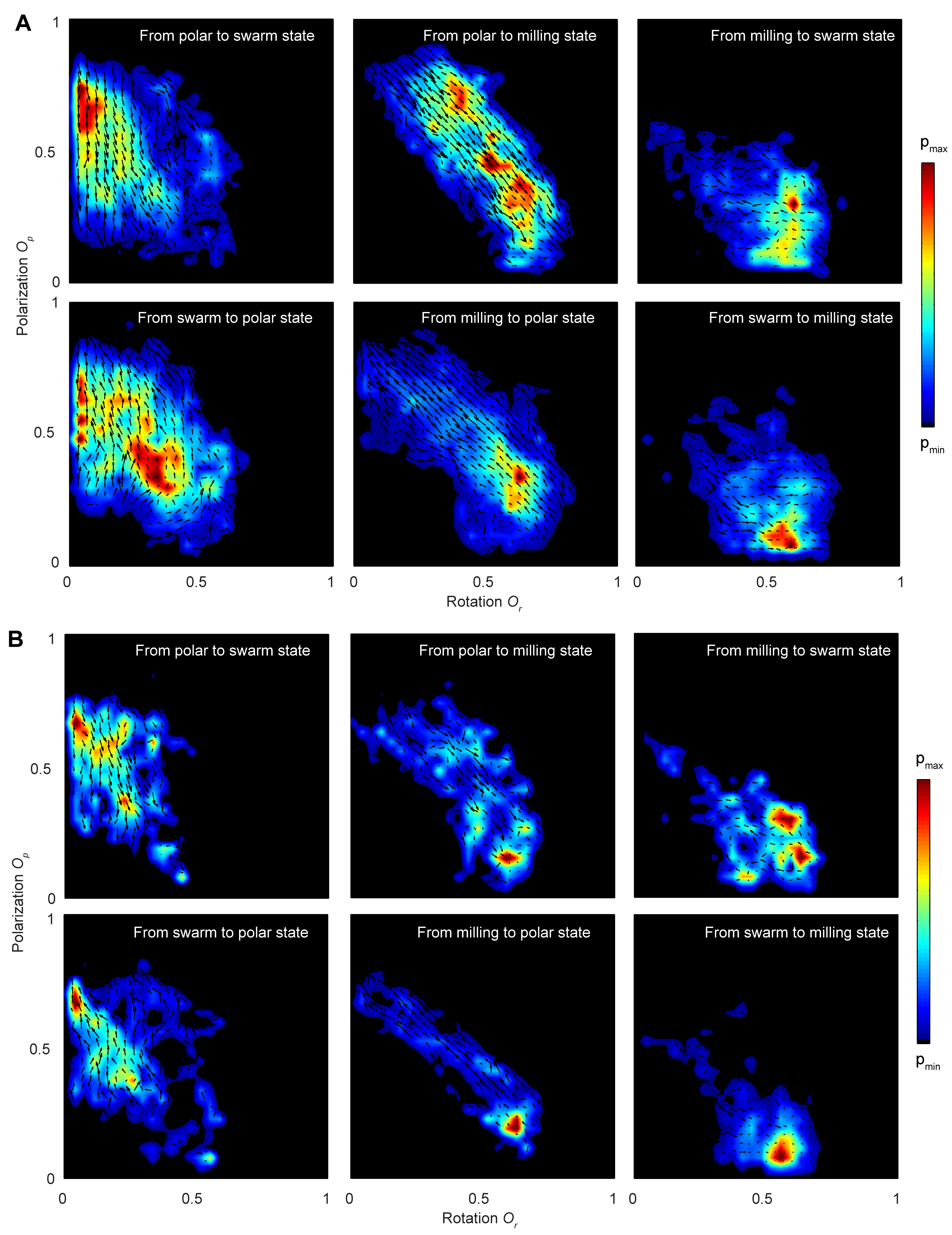

Supplement: Figure S7 — Transition patterns. Density plots of transitions between states for (A) 150 fish and (B) 300 fish. Overlaid the density plots are the corresponding velocity fields of the transition data (in the rotation-polarization phase space). (TIF) [file pcbi.1002915.s007.tif]

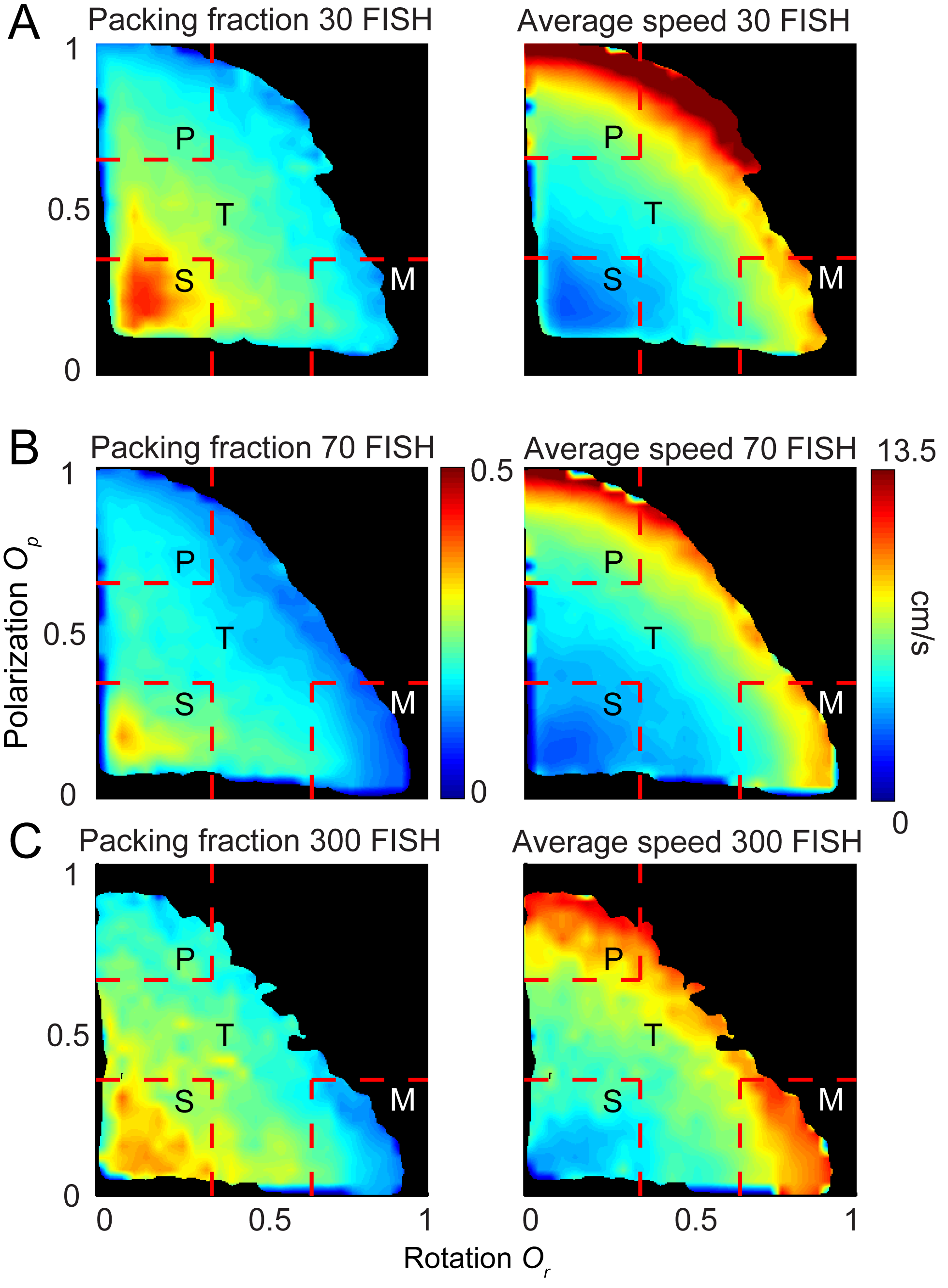

Supplement: Figure S8 — Packing fraction and average speed. Density plots of packing fraction and average individual speed (averaged per frame) as functions of rotation Op and polarization Or for (A) 30 fish, (B) 70 fish and (C) 300 fish. (TIF) [file pcbi.1002915.s008.tif]

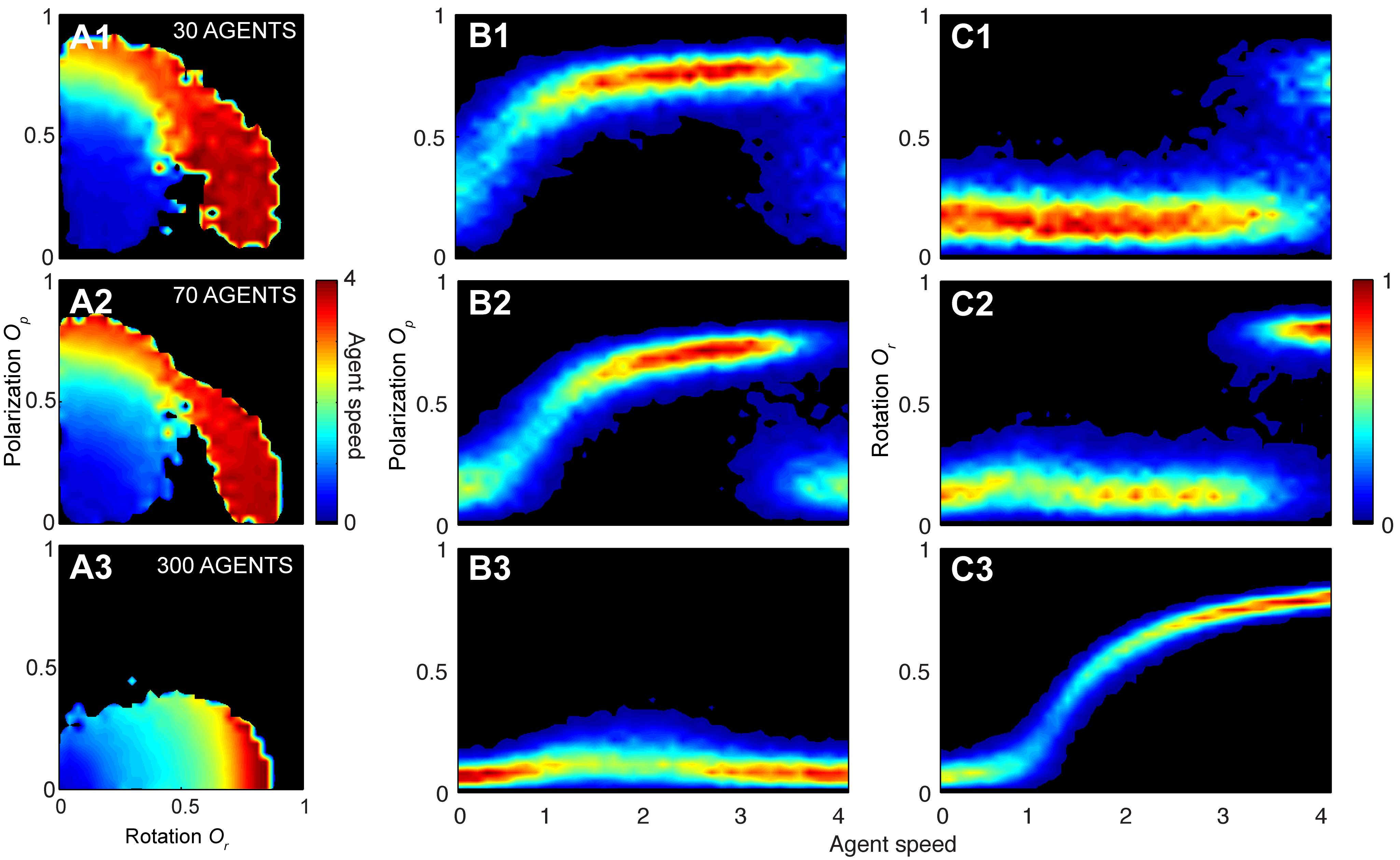

Supplement: Figure S9 — Relationship between agent speed and order in constant-speed agent based simulation model with 30, 70 and 300 agents. (A) Density plot of agent speed as function of rotation Or and polarization Op, revealing a bistable regime between the milling and the polar states for high speeds in simulations with 30 and 70 agents. For 300 agents the milling state becomes dominant and the bistable regime dissappears. (B) Normalized probability plot of polarization Op as function of agent speed. (C) Normalized probability plot of rotation Or as function of agent speed. (See Methods for simulation details). (TIF) [file pcbi.1002915.s009.tif]

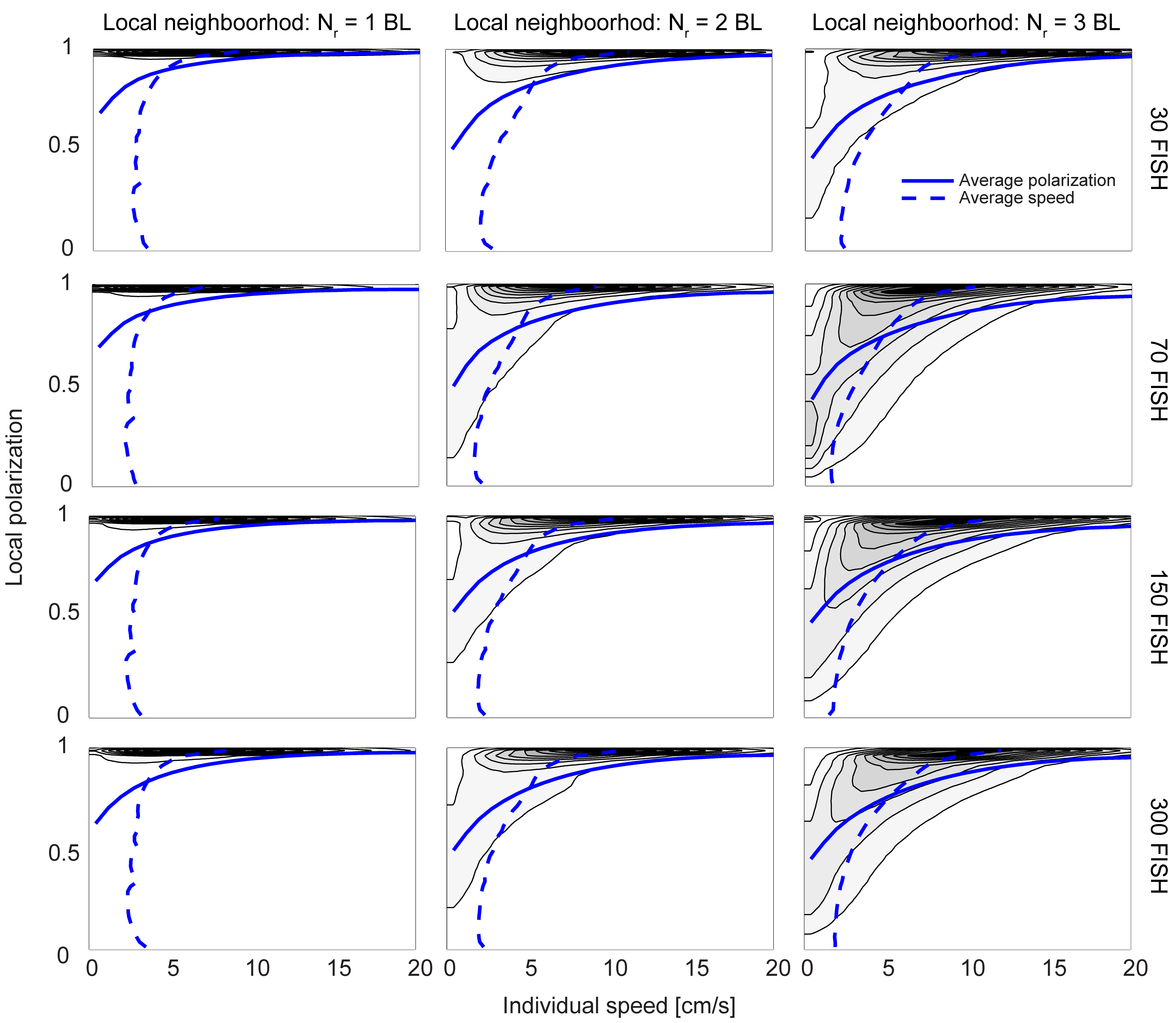

Supplement: Figure S10 — Relation between local polarization and individual speed. The plots show the correlation between individual speed and local polarization estimated in two ways from the underlying density maps. The stapled curves are produced by averaging across individual speeds for each value of the order parameter; the solid curves from averaging across the order parameter values for each individual speed. The local polarization of an individual fish is defined as the polarization Op restricted to the area inside a circle centered at the individual fish. The plots show the results from using neighborhoods of 1–3 body lengths (BL), where BL is 5.2 cm. (TIF) [file pcbi.1002915.s010.tif]

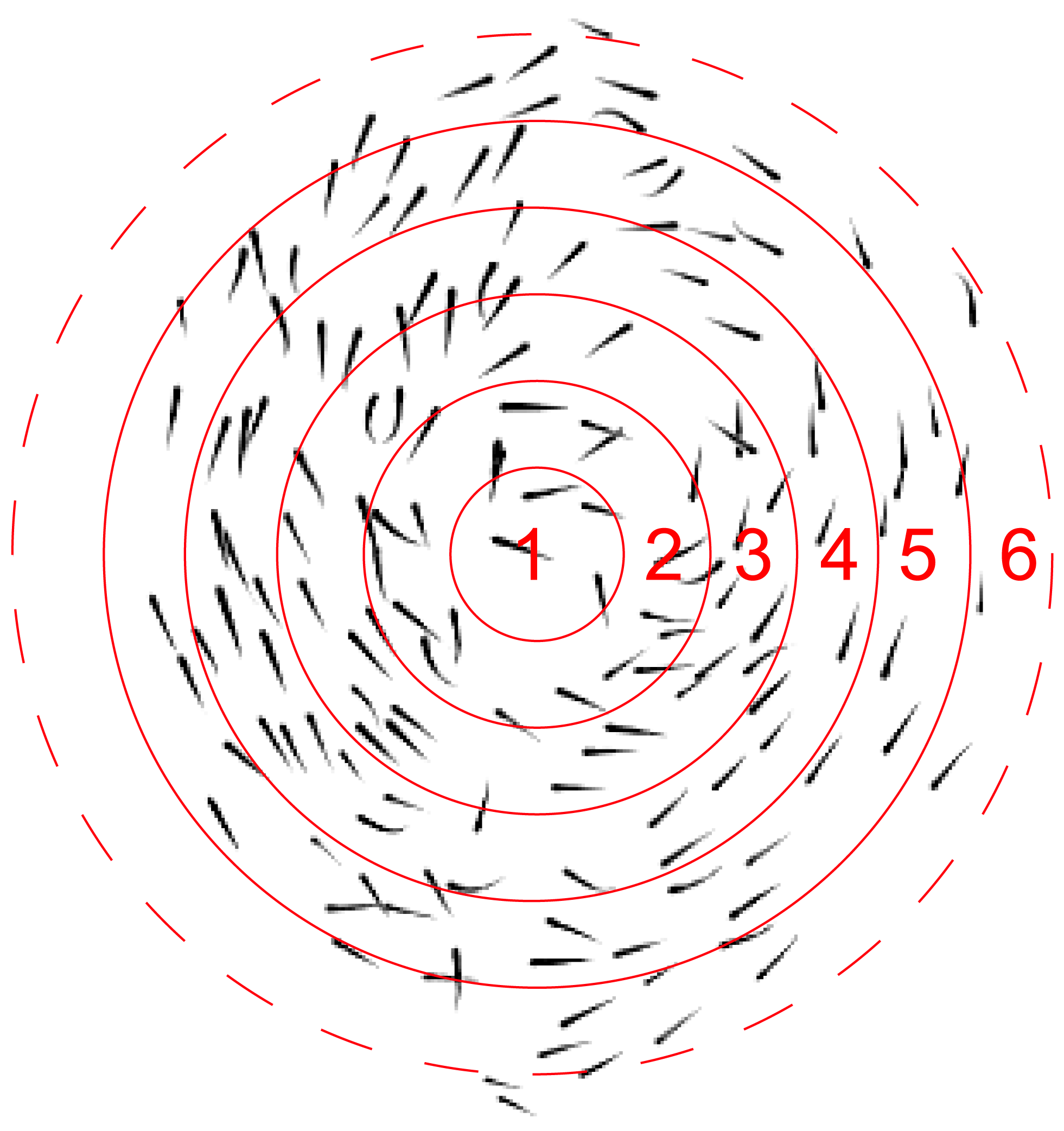

Supplement: Figure S11 — Radial division of milling state. The figure shows the division of fish in the milling state into shells, where the distance of the outer shell (dashed lines) is defined as the median distance to the group's centre of mass of the five most peripheral fish, and the width of each shell is the radius of the outer shell divided by six. (TIF) [file pcbi.1002915.s011.tif]

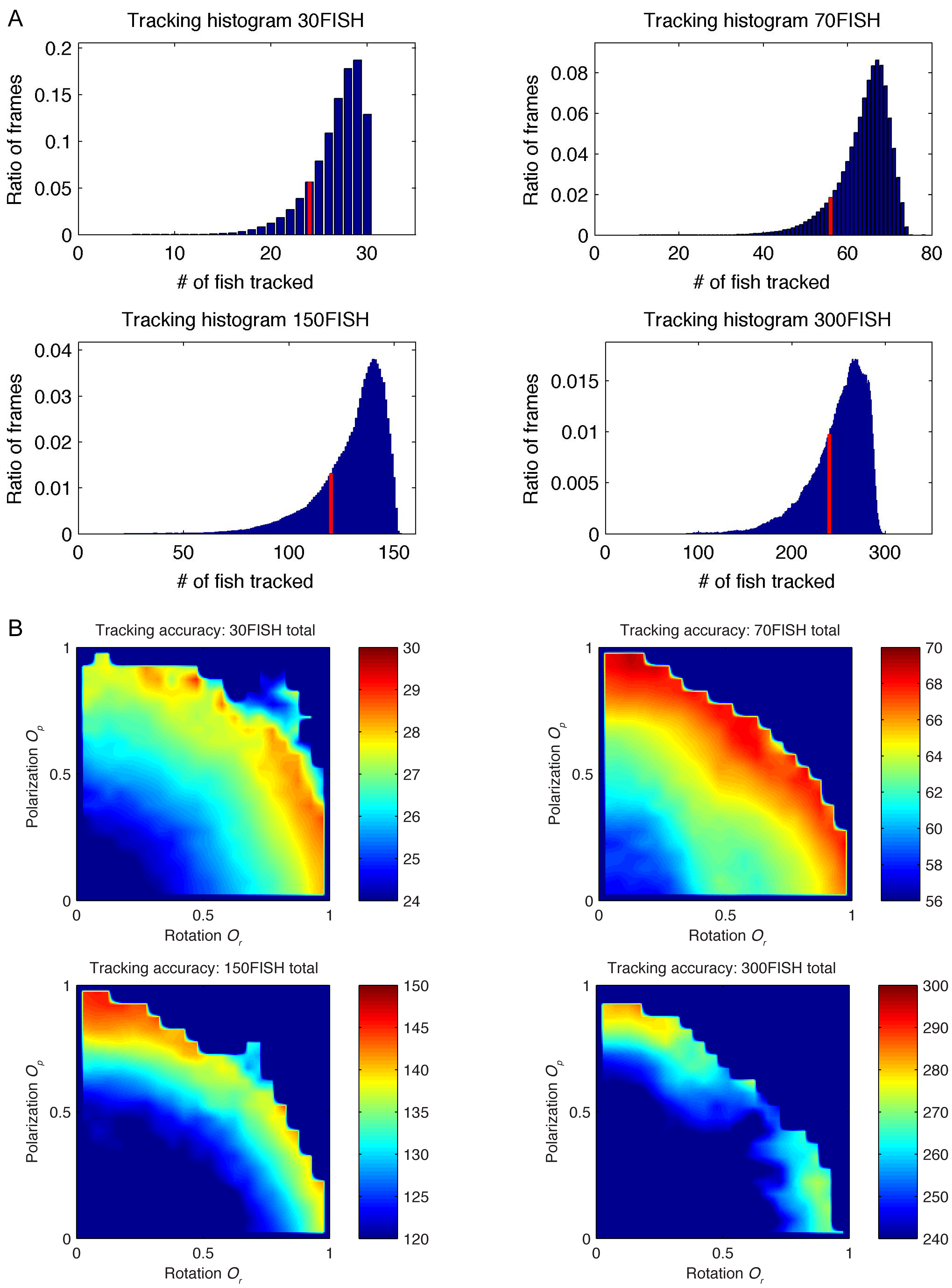

Supplement: Figure S12 — Tracking accuracy. (A) shows histograms of how many fish are tracked in each frame. The red line in each histogram denotes the threshold for 80% tracking accuracy. The percentage of frames above 80% accuracy are 88% for 30 fish, 91% for 70 fish, 80% for 150 fish and 71% for 300 fish. (B) shows the density distributions of tracking accuracy as a function of the rotational (Or) and polarization (Op) order parameters. (TIF) [file pcbi.1002915.s012.tif]
